# EFFECTIVENESS OF RADIAL EXTRACORPOREAL SHOCKWAVE THERAPY IN THE LOCAL MANAGEMENT OF HYPERTONIA (SPASTICITY AND DYSTONIA) IN PATIENTS WITH CEREBRAL PALSY

**DOI:** 10.2340/jrm-cc.v7.41084

**Published:** 2024-10-30

**Authors:** Tamara BIEDERMANN VILLAGRA, Miriam TUR SEGURA, Francisca GIMENO ESTEVE, Jordi JIMENEZ REDONDO, Nicolás GARCÍA RODRÍGUEZ, Raimon MILÀ VILLARROEL

**Affiliations:** 1 Physical Medicine and Rehabilitation Department, Fundació ASPACE Catalunya, Barcelona, Spain; 2 Research Comission, Fundació ASPACE Catalunya, Barcelona, Spain; 3 Neurorehabilitation Department, Fundació ASPACE Catalunya, Barcelona, Spain; 4 School of Health Sciences Blanquerna, Ramon Llull University, Barcelona, Spain

**Keywords:** cerebral palsy, extracorporeal shockwave therapy, hypertonia, spasticity

## Abstract

**Objective:**

To assess the effect of radial extracorporeal shockwave therapy on the reduction of local hypertonia in patients with cerebral palsy.

**Design:**

Explorative pre-post intervention study.

**Subjects/Patients:**

Forty-five patients with cerebral palsy.

**Methods:**

All patients received 3 sessions of radial extracorporeal shockwave therapy with a time interval of 1 week for each session. The outcomes were V1 and V3 of the Tardieu scale, the Timed Up and Go test, and the 10-metre walk test. The measurements were collected at baseline, immediately after the last session of shockwaves, at 12 and 24 weeks after baseline.

**Results:**

The statistical analysis used was a mixed linear model of repeated measures. The degrees on the Tardieu scale increased significantly in all the treated muscles. The results of the Timed Up and Go test and the 10 m walk test confirmed a significant functional effect after the shockwave therapy (*p* < 0.001).

**Conclusion:**

Functional improvement in patients treated with extracorporeal shockwave therapy has been observed to last up to 24 weeks.

## LAY ABSTRACT

Spasticity is the most prevalent motor disorder in patients with cerebral palsy. The objectives of its therapeutic approach include reducing pain, facilitating the use of orthopaedic aids, improving posture, minimizing contractures and deformities, enhancing mobility and dexterity, with the ultimate goal of maximizing the patient’s potential and promoting their independence and quality of life. The treatment with radial extracorporeal shockwaves represents a relatively new alternative to conventional approaches, and it has become established in recent years as a non-pharmacological and non-invasive option with few side-effects for the management of spasticity. According to current literature, the effect of radial Extracorporeal Shock Wave Therapy has not been investigated beyond 12 weeks.

Cerebral palsy (CP) is a leading cause of childhood motor disability, characterized by persistent movement and posture disorders due to early brain development issues. Its prevalence is 1.5 to 2.5 cases per 1000 live births (1, 2). Most individuals with CP have spasticity, which, along with other motor deficits, can cause pain, contractures, and skeletal deformities (1, 3, 4). Increased muscle tone hinders walking and orthotic use, increasing energy expenditure. In children, spasticity impedes muscle growth, raising the risk of contracture (5). Treatments for spasticity include physiotherapy, pharmacotherapy, orthopaedic interventions, and surgery (6).

Extracorporeal Shock Wave Therapy (ESWT) has demonstrated efficacy in treating various musculoskeletal and neurological conditions. It involves a sequence of single sonic pulses with high peak pressures, rapid pressure rise, and short durations through a generator to target specific areas, producing a physiological effect (7). Numerous studies have shown ESWT’s beneficial effects in managing spasticity in patients with conditions such as CP, multiple sclerosis, traumatic brain injury, and stroke (8–11). The exact mechanism of action of ESWT is still being investigated, but current research suggests it involves a cascade triggered by mechano-transduction: mechanical energy induces changes in cellular structures, prompting cellular responses that enhance processes like mitochondrial function, endoplasmic reticulum activity, and intracellular vesicle dynamics, ultimately improving healing (12).

The mechanism by which ESWT induces relaxation of spastic muscles remains incompletely understood, but hypotheses suggest several pathways. ESWT may induce rheological effects, altering muscle elasticity and extensibility. Moreover, it is proposed that ESWT stimulates nitric oxide production, influencing neuromuscular junctions and modulating interleukin secretion (13). Additionally, the application of ESWT to spastic muscles appears to transiently disrupt nerve conduction at neuromuscular junctions (14).

There are 2 modalities of shockwave therapies: focused ESWT (fESWT) and radial (rESWT). These 2 types of ESWT differ in physical properties, generation modes, magnitudes of standard parameters applied, and depths attained. The pressure produced by the fESWT rapidly increases, and energy is absorbed as deep as 12 cm. The pressure produced by rESWT increases much more slowly than that produced by focused ESWT, and the depth of energy is shallow at only 3–4 cm (10, 15).

The aim of this study was to evaluate the physiological and functional changes of the person after performing rESWT for spasticity.

## METHODS

A pre-post study was conducted to evaluate the intensity and duration of the effect of radial shockwave treatment in the local management of hypertonia in patients with CP. This study was approved by the Clinical Research Ethics Committee (CEIC-CEI) FIDMAG (Barcelona) with registration number PR-2021-21 conducted in accordance with the Helsinki Declaration of the World Medical Association.

### Patients

Inclusion criteria comprised individuals of any sex, aged 6 years or older, diagnosed with spastic CP, and who provided signed informed consent, either directly or through a legal representative. Exclusion criteria included systemic rheumatic conditions, associated neuromuscular disorders, recent orthopaedic lower limb surgery within the past 6 months, inability to comply with treatment, recent botulinum toxin injections or shockwave therapy to the lower limbs within the last 6 months, significant skin abnormalities in the treatment area, and any contraindications for shockwave therapy (e.g., infection or tumour at the therapy site, severe blood disorders, ongoing anticoagulant therapy).

### Methods

After obtaining informed consent from participants or their legal representatives, baseline data collection included age, sex, type of CP, and classification according to the Gross Motor Function Classification System (GMFCS) guidelines from the European Cerebral Palsy Register.

All subjects received 3 sessions of rESWT with a time interval of 1 week between each session using the Swiss Dolor Clast Smart model with the Evo Blue probe from EMS (Electro Medical System), Nyon, Switzerland. Treatment parameters were standardized: 2000 impulses were applied using a 15 mm applicator at 2.2 Bars and 8 Hz frequency for the calf muscles (Triceps Surae and Soleus), and a 36 mm applicator at 3.5 Bars and 8Hz frequency for the hamstring muscles. Conductive gel was applied, and a sweeping motion covered the entire treatment area, with each muscle group treated for 4 min. Since all patients who participated in the study were sourced from our foundation, where they regularly undergo physical therapy sessions, and the primary objective of the study was to assess the effect of shockwave treatment within their usual therapeutic approach, all patients continued their physical therapy sessions tailored to their specific needs. The therapy hours and the types of exercises performed were recorded. All patients averaged 1 h of physical therapy per week.

The primary study variable was the physiological response of treated muscles, evaluated by assessing resistance to slow (V1) and fast (V3) passive movements on the Tardieu scale (16). V1 measured the initial muscular reaction angle at low velocity, equivalent to the passive range of motion (pROM). V3 measured both the reaction angle and quality at maximum velocity. All measurements were performed with an inclinometer. For the measurement of calf muscles, separate stretches were applied to the gastrocnemius muscle (measured with the knee extended) and the soleus muscle (measured with the knee flexed at 90°). For hamstring muscles using the popliteal angle starting from 90 degrees of hip and knee flexion.

As a secondary variable, functional improvement in overall mobility was assessed using the Timed Up and Go test (TUG), measuring the time taken to rise from a chair, walk 3 m, return, and sit down (17). Gait improvement was evaluated with the 10-Metre Walk Test (10-MWT), recording the time taken to walk 10 m in a straight line (18). Both tests were conducted twice, and the best time from each was recorded.

Data collection occurred at baseline (T0), immediately post-treatment (T1), at 12 weeks (T2), and at 24 weeks (T3) after baseline.

### Statistical analysis

A mixed linear model was employed, with the fixed factors being the physiological and functional assessments at the 4 study time points (T0, T1, T2, T3), considering subjects as random factors. The results were analysed using IBM SPSS Statistics v29 software. The significance level was set at *p* ≤ 0.05.

## RESULTS

A total of 45 patients were recruited, 23 were men (51,5%) and 22 (48,9%) were women, with ages ranging from 8 to 56 years (mean age for men: 29.9; mean age for women: 21.2; only 9 patients < 18). According to the GMFCS classification, 14 were classified as GMFCS-I (31.1%), 19 as GMFCS-II (42.2%), and 12 as GMFCS-III (26.7%). Regarding the topographic distribution of the injury, 22 had hemiplegia (48.9%), 12 had diplegia (26.7%), 10 had quadriplegia (22.2%), and 1 had paraparesis (2.2%).

The muscles treated with rESWT were as follows: soleus-gastrocnemius (triceps surae) in 86.6% (39) of patients; hamstrings in 28.8% (13); and adductors in 15.5% (7).

### Physiological results

It should be noted that 22 patients have hemiplegia, and therefore, the number of values for the right and left muscles does not correspond to the total number of patients. Tables I and II present the sample size (N) for each muscle, their degree values at different time points during the study, and the statistical significance (p) obtained from the intersection of the mixed linear model. Table I reports the results for the passive range of motion (V1, Tardieu Scale), while Table II displays the results for the stretch reflex (V3, Tardieu Scale).

**Table I:** Passive range of motion (V1 Tardieu Scale)

Passive range of motion V1 Tardieu Scale							
		*N*	T0	T1	T2	T3	*p*
Gastrocnemius	RIGHT	24	3.1	11	13.3	12.3	< 0.001
	LEFT	23	7.4	14.1	15.1	12.2	< 0.001
Soleus	RIGHT	24	13.5	22.5	22.2	21.7	< 0.001
	LEFT	23	18.7	26.1	25.8	22.6	< 0.001
Hamstrings	RIGHT	10	27.0	37.0	38.9	33.5	< 0.001
	LEFT	10	32.5	42.5	38.3	38.0	< 0.001

**Table II:** Stretch Reflex (V3 Tardieu Scale)

Stretch Reflex V3 Tardieu Scale							
		*N*	T0	T1	T2	T3	*p*
Gastrocnemius	Right	24	–10.0	-0.2	1.1	-0.8	0.643
	Left	23	–8.0	2.0	1.2	0.2	0.907
Soleus	Right	24	–2.7	6.5	6.7	6.0	0.003
	Left	23	–3.9	6.3	4.5	2.4	0.248
Hamstrings	Right	10	3.5	16.0	18.3	15.0	< 0.001
	Left	10	7.0	17.5	17.2	17.5	< 0.001

### Functional test results

The results of the mixed linear model for the TUG test display the time values, in seconds, that each patient required to complete the 6-m test, including the time to rise and sit back down.

The results of the mixed linear model for the 10-MWT show the velocity (m/s) at which each patient covered the 10-m distance (Table III).

**Table III:** Time in the Timed Up & Go Test and velocity in the 10-Metre Walk Test

	*N*	T0	T1	T2	T3	*p*
Timed Up&Go Test (s)	44	9.50	8.09	7.60	7.45	< 0.001
10-Metre Walk Test (m/s)	45	1.39	1.59	1.67	1.66	< 0.001

## DISCUSSION

### About physiological results

The findings of this study indicate an improvement in the physiological data of muscles treated with rESWT, observable immediately after the final rESWT session (T1).

In Wang’s study, spasticity was assessed using the modified Ashworth scale and pROM. A statistically significant improvement in pROM was found before and after treatment. Both pROM and the V1 component of the Tardieu scale were measured in the same manner: dorsiflexion of the ankle joint was evaluated using a goniometer in a supine position, both prior to and following treatment with rESWT.

In our study, a statistically significant increase in the degrees of extensibility (V1) of all treated muscles was observed at T1 and T2, with a progressive decline at T3 that did not return to baseline values (T0). This trend of maintaining a residual positive effect on muscle extensibility aligns with the findings reported by Vidal et al. (19), where the effect had not completely diminished even 6 months after the initiation of rESWT treatment.

Regarding the results obtained in the assessment of the muscle reaction angle at high speed (V3), there is evidence of more pronounced variability and a lack of significant improvement in the triceps surae and soleus muscles. However, an improvement in V3 was observed in the hamstrings. Overall, the stretch reflex response appears to be more variable across the different muscle groups evaluated.

Although it has been documented that spastic muscles in CP tend to be shorter and smaller in size, it is important to note that the muscle fibre structure exhibits atypical characteristics. These muscle fibres are reduced in size, contain fewer sarcomeres, and are more elongated than those observed under normal conditions. This phenomenon leads to pathological muscle adaptation. Additionally, an increase in the extracellular matrix and a decrease in the satellite cell population have been observed in these muscles (20). The effect of radial shock wave therapy on spastic muscle is also uncertain, although some studies suggest that: (i) it induces a cascade of biological responses, such as the expression of growth factors related to angiogenesis, which produce an antifibrotic effect by increasing blood flow and facilitating tissue regeneration; (ii) it induces the production of nitric oxide, which modulates neurotransmission at neuromuscular junctions; (iii) it induces the degeneration of acetylcholine receptors; and (iv) it can selectively destroy the terminal plates of neuromuscular junctions. These processes could explain the observed effects on the improvement of muscle extensibility induced by radial shock waves in spastic muscle.

### About functional results

**Fig. 1:**
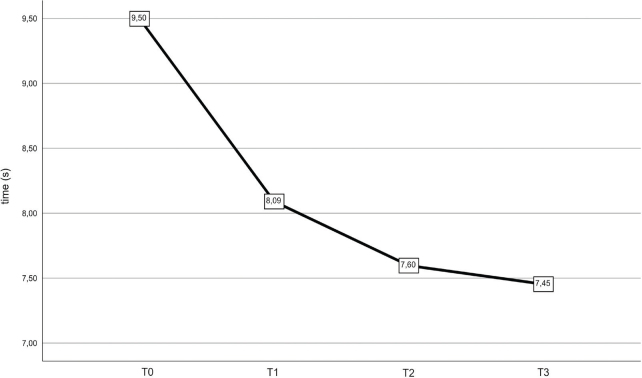
Time (seconds) in the Timed Up & Go Test from T0 (baseline) to T3 (24 weeks after).

**Fig. 2:**
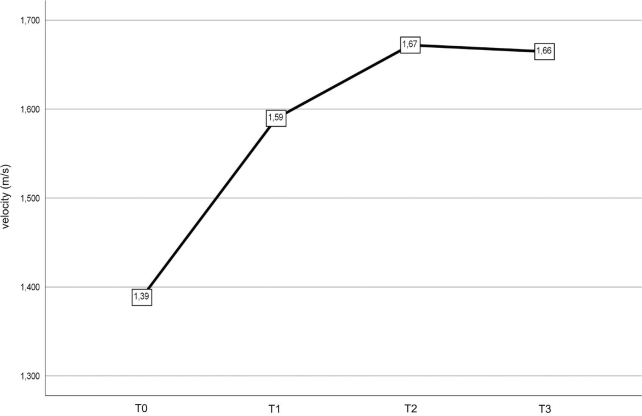
Velocity (m/s) in the 10-Metre Walk Test from T0 (baseline) to T3 (24 weeks after).

The overall mobility of patients, assessed with the TUG test, improved at T1 and was maintained at T2 and T3, as shown in Fig. 1. Similarly, gait assessment with the 10-MWT demonstrated improvements, as depicted in Fig. 2.

The improvement observed in gait speed and stability may be linked to the increase in muscle extensibility. According to the findings reported by Lin et al. (21) and Amelio et al. (22), the application of ESWT to the triceps surae muscle resulted in increased muscle extensibility at the ankle joint, as well as a significant increase in surface area and plantar pressure. These findings suggest a correlation with the observed improvement in gait parameters and overall stability in the patients analysed in this study.

We have not found other studies on individuals with CP that investigate changes in motor functionality following rESWT treatment (such as gait speed and TUG test performance), so we cannot compare these results.

The main hypothesis for these results is that the cavitation produced in the muscles treated with radial shock waves has led to a reduction in muscle tone. This is explained by mechanotransduction, as described by Jaalouk et al. (23), which triggers a biological response in the stimulated tissues. This response promotes tissue regeneration and angiogenesis by increasing metabolism and vascularization in the impacted areas, as well as analgesia through the destruction of cell membranes of pain receptors and the release of endorphins and other analgesic substances, as noted by Contaldo et al. (24).

The extent to which improvements in controlled functional tests translate to daily life remains uncertain. Motor performance times in specific tests may improve without substantial enhancement in overall daily functional capacity. To explore this, a qualitative study analysing treated patients’ subjective perspectives via semi-structured interviews has been conducted (currently in press).

To sustain these functional improvements, treatment continuation beyond 6 months, involving 3 sessions of rESWT with a time interval of 1 week between each session, has been deemed necessary. Future studies should investigate whether adjusting treatment frequency enhances functional response and maintains shock wave effects beyond 6 months.

Due to ethical considerations, this study did not include a control group receiving no treatment. Consequently, the results only reflect responses from treated patients, without randomization or blinding during data collection. The study aimed to observe shock waves physiological and functional effects and their duration, not to compare treatments. Muscle angles were measured using a manual inclinometer, possibly introducing deviations in pROM data. Timing relied on a handheld stopwatch, which could also introduce measurement errors. Nevertheless, we believe these errors are smaller than the observed improvements. All measurements were performed by the same individuals, using the same instruments, and in the same location within the centre. Given that the study design did not allow for evaluator blinding, the following measures were taken to minimize bias: once a measurement was taken, it was recorded and handed over to a member of the research team, who archived it in the patient’s dossier. The individuals responsible for the measurements did not have access to previous measurements. Overall, results indicate functional improvement in treating spastic CP with a non-invasive technique, showing minimal risks and no significant side-effects. Comparable studies focusing on functional motor outcomes following shock wave treatment in individuals with CP were not found in published literature.
